# Supplementary material for: Genetic Mutations Associated with Inflammatory Response Caused by HPV Integration in Oropharyngeal Squamous Cell Carcinoma
Source: Biomedicines. 2023 Dec 21;12(1):24. doi: 10.3390/biomedicines12010024 (PMC10813733; doi:10.3390/biomedicines12010024)
Supplement: Supplementary file 1 [file biomedicines-12-00024-s001.zip › biomedicines-2759319-supplementary.pdf]

Supplemental Tables & Figures

Supplemental Table S1: Data extraction of the included articles in the scoping review. The 38 articles present the list of HPV-positive (+) and HPV-negative (-) mutated genes. 2 articles out of the 38 articles screened have included both lists of genes. However, the rest included HPV + mutated genes which this scoping review mainly highlights. 330 genes were identified and submitted to enriched analysis. *TP53* (n= 22) and *PIK3CA* (n= 20) genes were the most mutated genes in HPV-related OPC cases.

AUTHOR, YEAR	HPV + GENES MUTATED	HPV - GENES MUTATED
1. R. Alex Harbison et al., 2018	KMT2D, FGFR3, CYLD, EP300, PIK3CA, RB1, PEG3, STAT3, TTSC2, B2M, CREBBP, FBXW7, FLT1, NCOR1, NSD1, PTEN, USP9X, BRIP1, NBN, NFE2L2, TACC3, ARID1B, ARID5B, DDR2, EPHA2, FANCA, KDM5C, LRRK2, MAP2K2, MAPK1, NOTCH3, PTPRD, SMAD2, SYK, TRAF3, TRRAP. FLT1, IDH2, AR, ASXL1, ATM, AXIN1, BAP1, BCR, CIC, ELF3, FANCA, FAT1, FLT4, GRIN2A, HDAC4, HIF1A, IFNGR1, KMT2D, LRP2, MAP3K5, MED12, MTOR, NBN, PIK3R6, RB1, SRC, TACC3, TRRAP, TSC2, XPO1.<br>**RECURRENT OPSCCS:-** TP53, CASP8, FAT1, HLA-A, AJUBA, AND NSD1.	
2. Chung, C. H. et al, 2015	IK3CA, SOX2, MLL2 (KMT2D), RB1, BCL6, EP300, NOTCH1, PTEN, FGFR3, ASXL1, KLHL6, FBXW7, TP53, ATM, BRCA2, BRIP1 (BACH1), LRP1B, ATRX, KDM6A, BRCA1, BLM, JAK2, NF1, HRAS, MYC, ATR, FGF19, FGF3, FGF4, RICTOR	TP53, CDKN2A/B, FGF19, FGF3, FGF4, PIK3CA, CCND1, NOTCH1, LRP1B, SOX2, MLL2 (KMT2D), EGFR, KLHL6, BCL6, ATR, NFE2L2, NOTCH2, MYC, FGFR1, ATRX, JAK2, SMAD4, RICTOR, ZNF703, BRCA2, FOXL2, PRKDC, GPR124, KDM6A, APC.
3. Doerstling, S. et al., 2023	ATM, CCND1, CDKN2A, RB1, EGFR, FBXW7, FGFR1, FGFR2, FGFR3, IDH1, KRAS, NRAS, HRAS, NOTCH1<br>AKT1, MTOR, PIK3CA, PTEN, TP53, AR, ALK,, BRAF, BRCA1, BRCA2, CDK/RB PATHWAY (CCND1, CDKN2A, RB1), FGFR1-4, FLT3, JAK2, MET, MLH1, MSH2, MSH6, PI3K PATHWAY (AKT1, MTOR, PIK3CA, PTEN).	
4. Dogan, S. et al., 2019	TP53, SOX2, CDKN2A/2B, PIK3CA, TP63, KMT2D , NOTCH1, FAT1, 11Q13 GENE CLUSTER (FGF3/FGF4/FGF19/CCND1) , FOXA1, NOTCH PATHWAY GENES (NOTCH1, NOTCH2, NOTCH3, NOTCH4, EP300, FBXW7, SPEN, KDM5A), HISTONE MODIFIERS (KMT2D, CREBBP, KMT2C, EP300, KMT2A). NFE2L2, KEAP1, CUL3, EGFR, ERBB2, FGFR1, FGFR3, FOXA1, TERT, NKX2-1, FGFR1, PMA1P1, ERBB2, EGFR, YAP1, ATM, MYC, KEAP1, CASP8, CUL3. JAK3. MAP3K13, FOXA1, HRAS, CCND1,PIK3/AKT/MTOR PATHWAY. FAT1, RUNX1.	
5. Dubot, C. et al., 2018	CDKN2B, RB1,MDM2, RICTOR, PI3K, KRAS, NRAS, MAPK, AJUBA, SYNE2, USP9X, KDM6A, NSD1, LRP1B. CELL CYCLE PATHWAY (TP53, CCND1, CDKN2A), PI3K/AKT/MTOR PATHWAY (PIK3CA), TYROSINE KINASE RECEPTORS (EGFR, FGFR1), CELL DIFFERENTIATION (FAT1, NOTCH1).	
6. Gleber-Netto, F. O. et al., 2018	TP53, NOTCH1, CDKN2A, NOTCH2, PIK3CA, FAT1, FBXW7, KEAP1, NFE2L2, NSD1, TP63, EGFR, HRAS, CASP8, CCND1, TGFBR2	
7. Gronhoj, C. et al., 2018	APOB, BIRC6, SPTBN1, FAT2, KMT2A, FAT1, BPTF, TRIO, HERC2, KALRN, ZNRF3, BNC2, NOTCH2, FGFR2, SMAD2, AR, SIN3A, PTCH1, DNMT3A, ARHGAP35, F5, IGF1R, CATSPER1, IQGAP1, SFMBT2, MET, DUBP1, TENM2, TSC1, ARID5B, FAT2, FNDC1, BIRC6, PTPN14, QSER1, ALS2CL, PIK3CB, ARID2, NOTCH3, APOB, FGFR1, NF2, NRXN3, MYH9, PRPF8, FOXP1, PEX2, PBRM1, IPO7, SPTA1, TRIO, ABCG1, TJP2, EP300, RET, SLX4, AKT2, FN1, HCFC1, PCDH18, WHSC1, BPTF, CREBBP	
8. Haft, S. et al., 2019	PIK3CA, KMT2C, FBXW7, FGFR3, CREBBP, FAT1, NSD1, KMT2D, NOTCH1, CASP8, CLTCL1, EPHA2, HLA-A, HRAS, PDE4DIP, PTCH1, PTEN ZNF750, ADCY4, AJUBA, ATM, CTCF, CUL3, FANCA, MET, LRP1B, CASZ1, CYLD, EP300, KALRN, MACF1, ASXL3, CSMD3, DST, FLNC, HUWE1, KIAA1407, ASPM, DNAH5, FAM135B, HERC1, HFM1, LRRC37B, MAP1B, MUC4, MUC5B, POLR3A, TRAF3, SERPINB5, USP6, FLG	
9. Koncar, R. F. et al., 2017	ABL1, AKT1, ALK, APC, ATM, BRAF, BRCA1, BRCA2, CDH1, C-KIT, CMET, CSF1R, CTNNB1, EGFR, ERBB2, ERBB4, FBXW7, FGFR1, FGFR2, FLT3, GNA11, GNAQ, GNAS, HNF1A, HRAS, IDH1, JAK2, JAK3, KDR, KRAS, MLH1, MPL, NOTCH1, NPM1, NRAS, PDGFRA, PIK3CA, PTEN, PTPN11, RB1, RET, SMAD4, SMARCB1, SMO, STK11, TP53, VHL.	
10. Labarge, B. et al., 2022	FLG, PIK3CA, MUC12, ZNF750, USE1, KMT2D, MUC6, TP63, SLITRK3, NLRC5, MORN1, EFNB2, HLA-A, KRTAP1-1, B2M, PXN, CDKN2A, RB1, TRAF3, PTEN.	
11. Lim, S. M. et al., 2019<br>Multicentric study	TP53, CDKN2A, CCND1, PIK3CA, KMT2C, FAT1, RELN, FAT4, CDKN2B, EGFR, KMT2D, NFE2L2, ADGRV1, NOTCH1, FAT2, CTTN, MYC, GNAS, EPHB4, ASXL3, CHD4, SOX2, ASXL1, KEAP1, AR, NOTCH2, KLHL6, TERT, KRAS, PTEN, MAP3K9, CDH9, CDH1, HPV	
12. Qin, T. et al., 2018	PDE4DIP, FAT1, NOTCH2, AHNAK, NUMA1, MKI67, ABCC3, ABCC1, PRKDC, TP53, MUC16, NRG1, TIAM1, NOTCH3, CASP8, CEP290, KLRC2, MAP3K1, NBAS, PTPRB	
13. Reder, H. et al., 2019	TP53, RB1, STK11, CDH1, HRAS, KRAS, NRAS, FAT1, PIK3CA, PIK3R1, PTEN, FANCA, FBXW7, CYLD, BCL6, TP63, TAF1, EP300, DDX3X, NOTCH1, JAK1, JAK2, PDGFRA.	
14. Reder, H. et al., 2021.	TP53, RB1, STK11, CDH1, HRAS, KRAS, NRAS, FAT1, PIK3CA, PIK3R1, PTEN, FANCA, FBXW7, CYLD, BCL6, TP63, TAF1, EP300, DDX3X, NOTCH1, JAK1, JAK2, PDGFRA.	
15. Saba, N. F. et al., 2020	PIK3CA, TP53, KMT2A, GNAQ, KDM6A, LAMA2, PTEN, DDX3X, BRCA1, BRCA2, ABCC2, CHD7, ERBB3, H3F3A, INPP4B, RB1, JAK2, NF1, PDGFRA, ALK, FGFR2, MAP2K2, MAPK1, MET, RET, ROS1, SOS1, FLT3, KIT, KRAS, PTEN, INPP4B, AKT1, AKT2, MTOR, PIK3R1, RPS6KB1	
16. Wahle, B. M. et al., 2022	PIK3CA, FGFR3, ZNF750, SYNE2, FLG, SYNE1, PLXNA1, PLEC, HERC1, ZFHX4, VPS8, USH2A, TRAF3, TACC2, RYR3, PTEN, OTOG, LAMA2, KIAA1109, JRK, FBN3, EP300, DNAH5, DNAH14,	

	CUX1, BIRC5, AK5, SMARCAL1, PIK3R1, IQCG, METTL24, FBXW7, B2M, NRAS, IFI27, HLA-B, FGF2, AKT1, FGF8	
**17. Stransky, N. et al., 2011**	TP53, CDKN2A, CASP8, FAT1, NOTCH1, PTEN, SYNE1, HRAS, PIK3CA, MED1, MLL2, TP63, IRF6, EZH2, SYNE2, NOTCH3, RIPK4, NOTCH2, DICER1, RB1.	
**18. Williams, E. A. et al., 2021**	PIK3CA, KMT2D, FBXW7, PTEN, KMT2C, TP53, BCL2L1, NOTCH1, RB1, HPV16	
**19. Antonsson, A. et al., 2016**	EVER1 & EVER2	
**20. Barten, M. et al., 1995**	P53 MUTATION	
**21. Benzerdjeb, N. et al., 2021**	TP53 MUTATIONS.	
**22. Chen, Z. et al., 2021**	FAT1 MUTATIONS.	
**23. Chiosea, S. I. et al., 2013**	PIK3CA, HRAS AND PTEN GENE MUTATIONS.	
**24. Ekalaksananan, T. et al., 2020**	P53 R282 GENE MUTATIONS.	
**25. Fallai, C. et al., 2009**	TP53 MUTATIONS.	
**26. Farnebo, L. et al., 2015**	DNA REPAIR GENES XPC, XPD, XRCC1, XRCC3 AND HPV, P53 MUTATIONS.	
**27. Hong, A. et al., 2016**	P53 MUTATIONS.	
**28. Cortelazzi, B. et al., 2015**	PIK3CA, PTEN, IGF1R, IGF1, IGF2, HER2, HER3.	
**29. De Carvalho, A. C. et al., 2019**	TP53 MUTATIONS.	
**30. Friedland, P. et al., 2012**	**EGFR, KRAS, BRAF.**	
**31. Ghosh, A. et al., 2013**	**PHF2 , FANCC, PTCH1 ALTERATIONS**	
**32. Gross, A. M. et al., 2014**	**TP53 MUTATIONS, CASP8**	
**33. Huang, C. et al., 2019**	NSD1, NOTCH1, TP53, CDKN2A, PIK3CA	
**34. Licitra, L. et al., 2006**	TP53 MUTATIONS.	
**35. Mazurek, A. M. et al., 2016**	KRAS, EGFR GENE MUTATIONS	
**36. Saba, N. F. et al., 2015 Pilot Study**	CDKM2C, SYK, WNT10B, CEBPA, MAP3K8, FGR, GATA1, OL4, PDGFA, CDKN2A, E2F2, TP53, PIK3CA, FGFR3, RB1, MET	
**37. Sewell, A. et al., 2014**	PIK3CA MUTATIONS	
**38. Shaikh, H. et al., 2021**	PIK3CA, KMT2D, TP53, LOH, KMT2C, CYLD, FBXW7, NOTCH1, RB1, PIK3CB, PTEN, B2M, NF1, ASXL1, KDM6A, PIK3R2, CHEK2, NSD1, FAT1, MAPK1, CREBBP.<br>TERT, TP53, PIK3CA, RB1, NOTCH1, ARID1A, NF1, KMT2D, FAT1, LOH, ASXL1, KMT2C, CDKN2A, FBXW7, ATM, FGFR3, KDM6A, FANCM, EP300, B2M, KRAS.	TP53, TERT, PIK3CA, NOTCH1, FAT1, CDKN2A, LOH, KMT2D, RB1, KMT2C, ASXL1, NF1, EP300, ARID1A, FBXW7, NFE2L2, CYLD, NSD1, KDM6A, PBRM1, FGFR3, HRAS.<br>TP53, TERT, CDKN2A, LOH, KMT2D, NOTCH1, PIK3CA, FAT1, ARID1A, ASXL1, KMT2C, NSD1, FBXW7, EP300, RB1, CREBBP, NF1, CYLD, KDM6A, HRAS, PTEN, NFE2L2.

Supplementary Table S2: Distribution of the OSCC cases according to demographic, lifestyle, and clinical variables.

Variable	Category	Paraffin-embedded samples n(%)	HELA2 n (%)		
			Negative	Positive	*P-value*
Age	< 50 year	8 (15.4)	7 (16.7)	1 (10)	0.599
	≥ 50 year	44 (84.6)	35 (83.3)	9 (90)	
Gender	Male	31 (59.6)	26 (61.9)	5 (50)	0.500
	Female	21 (40.4)	16 (38.1)	5 (50)	
Smoking habit	No	23 (44.2)	21 (53.8)	2 (22.2)	0.087
	Yes	29 (55.8)	18 (46.2)	7 (77.8)	
Alcohol consumption	No	16 (37.2)	13 (37.1)	3 (37.5)	0.985
	Yes	27 (62.8)	22 (62.9)	5 (62.5)	
Clinical stage	T1+T2	32 (61.5)	**29 (69)**	**3 (30)**	**0.023**
	T3+T4	20 (38.5)	**13 (31)**	**7 (70)**	
Lymph nodes	N0	40 (76.9)	34 (81)	6 (60)	0.158
	N+	12 (23.1)	8 (19)	4 (40)	
Recurrence or metastasis	No	40 (76.9)	31 (73.8)	9 (90)	0.275
	Yes	12 (23.1)	11 (26.2)	1 (10)	
Status	Alive	45 (86.5)	37 (88.1)	8 (80)	0.500
	Dead	7 (13.5)	5 (11.9)	2 (20)	

Supplemental Figure S1: shows list of all genes mutated with highlighting on the most cited genes which are *TP53* (n=22), *PIK3CA* (n=20), *PTEN* (n=16), *NOTCH1* (n=14), *RB1* (n=13), *FAT1* (n=13), *FBXW7* (n=12), *HRAS* (n=10), *KRAS* (n=10) and *CDKN2A* (n=10). Different colour codes representing the 38 articles screened to show which gene was collected from which article. The most frequently mutated gene is *TP53* followed by *PIK3CA*. Genes for cell survival and proliferation (*TP53*, *HRAS* & *PIK3CA*), cell-cycle control (*CDKN2A*), cellular differentiation (*NOTCH1*), and adhesion and invasion signalling (*FAT*). Tumor suppressor Genes (*FAT1*, *NOTCH1* & *CDKN2A*).

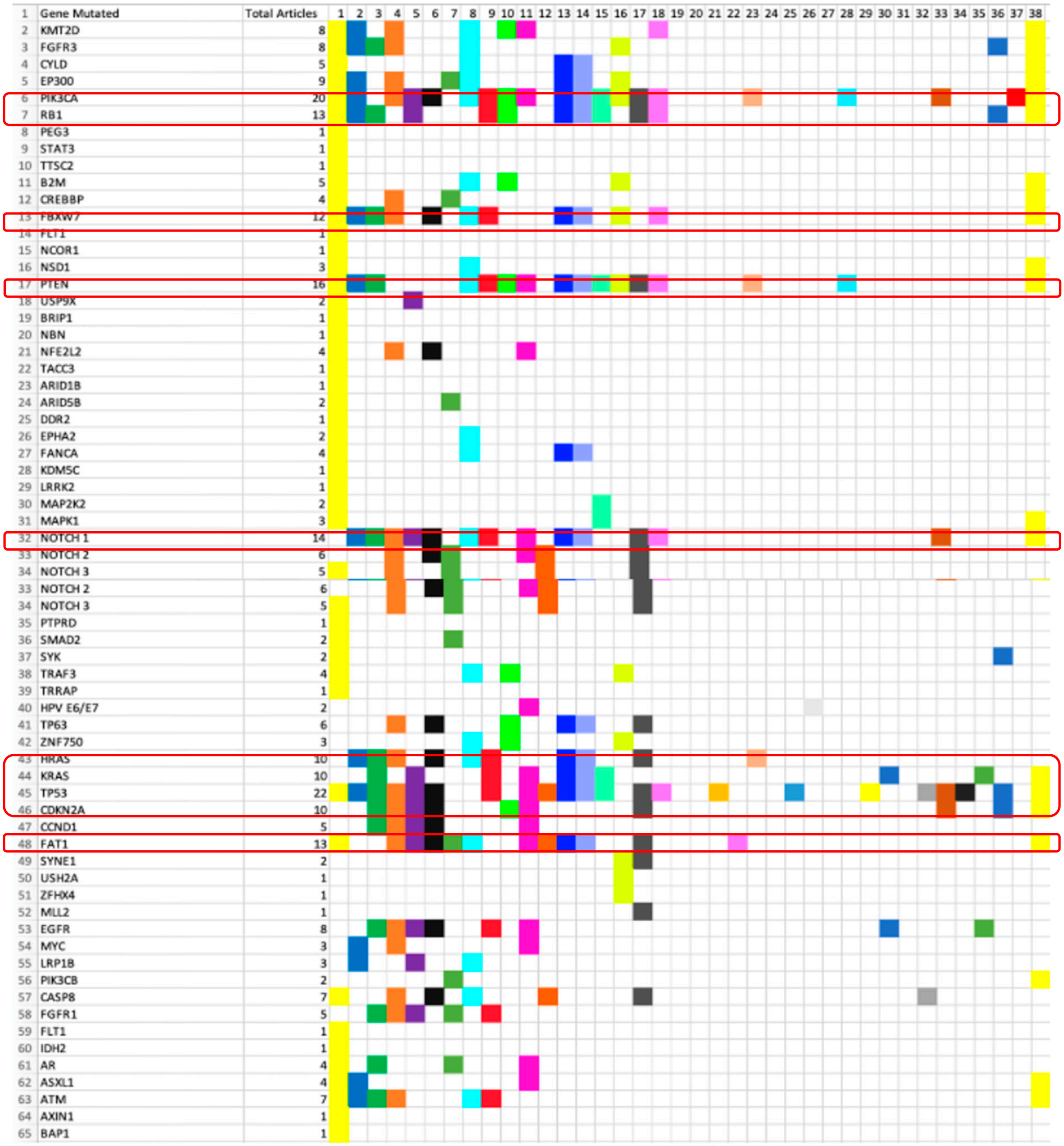

66	BCR	1
67	CIC	1
68	ELF3	1
69	FLT4	1
70	GRIN2A	1
71	HDAC4	1
72	HIF1A	1
73	IFNGR1	1
74	LRP2	1
75	MAP3K5	1
76	MED12	1
77	MTOR	3
78	NBN	1
79	PIK3R6	1
80	SRC	1
81	TSC2	2
82	XPO1	1
83	HLA-A	3
84	AJUBA	2
85	NSD1	4
86	BRIP1	1
87	STK11	3
88	PIK3R1	3
89	PIK3CA	2
90	SOX2	3
91	BCL6	3
92	KLHL6	2
93	BRCA2	4
94	ATRX	1
95	KDM6A	4
96	BRCA1	4
97	BLM	1
98	JAK2	6
99	NF1	3
100	ATR	1
101	FGF19	2
102	FGF3	2
103	FGF4	2
104	RICTOR	2
105	FGFR2	4
106	IDH1	2
107	NRAS	6
108	AKT1	4
109	ALK	3
110	BRAF	3
111	FGFR4	1
112	FLT3	2
113	MET	5
114	MLH1	2
115	MSH2	1
116	MSH6	1
117	RUNX1	1
118	CDKN2B	3
119	FOXA1	1
120	SPEN	1
121	KDM5A	1
122	KMT2C	5
123	KMT2A	3
124	KEAP1	3

125	CUL3	2
126	ERBB2	2
127	TERT	3
128	NKX2	1
129	NKX1	1
130	PMA1P1	1
131	YAP1	1
132	JAK3	2
133	MAP3K13	1
134	MDM2	1
135	PI3K	1
136	MAPK	1
137	SYNE2	3
138	TGFBR2	1
139	IQGAP1	1
140	APOB	1
141	BIRC6	1
142	SPTBN1	1
143	FAT2	2
144	BPTF	1
145	TRIO	1
146	HERC2	1
147	KALRN	2
148	ZNRF3	1
149	BNC2	1
150	SIN3A	1
151	PTCH1	3
152	DNMT3A	1
153	APHGAP35	1
154	F5	1
155	IGF1R	2
156	CATSPER1	1
157	SFMBT2	1
158	DUBP1	1
159	TENM2	1
160	TSC1	1
161	FNDC1	1
162	PTPN14	1
163	QSER1	1
164	ALS2CL	1
165	ARID2	1
166	NF2	1
167	NRXN3	1
168	MYH9	1
169	PRPF8	1
170	FOXP1	1
171	PEX2	1
172	PBRM1	1
173	IPO7	1
174	SPTA1	1
175	ABCG1	1
176	TJP2	1
177	RET	3
178	SLX4	1
179	AKT2	2
180	FN1	1
181	HCFC1	1
182	PCDH18	1
183	WHSC1	1
184	CLTCL1	1
185	PDE4DIP	2
186	ADCY4	1
187	CTCF	1

188	CASZ1	1
189	MACF1	1
190	ASXL3	2
191	CSMD3	1
192	DST	1
193	FLNC	1
194	HUWE1	1
195	KIAA1407	1
196	ASPM	1
197	DNAH5	2
198	FAM135B	1
199	HERC1	2
200	HFM1	1
201	LRRC37B	1
202	MAP1B	1
203	MUC4	1
204	MUC5B	1
205	POLR3A	1
206	SERPINB5	1
207	USP6	1
208	FLG	3
209	ABL1	1
210	APC	1
211	CDH1	4
212	c-KIT	1
213	cMET	1
214	CSF1R	1
215	CTNNB1	1
216	ERBB4	1
217	GNA11	1
218	GNAQ	2
219	GNAS	2
220	HNF1A	1
221	KDR	1
222	MPL	1
223	NPM1	1
224	PDGFRA	4
225	PTPN11	1
226	SMAD4	1
227	SMARCB1	1
228	SMO	1
229	VHL	1
230	MUC12	1
231	USE1	1
232	MUC6	1
233	SLITRK3	1
234	NLRC5	1
235	MORN1	1
236	EFNB2	1
237	KRTAP1-1	1
238	PXN	1
239	RELN	1
240	FAT4	1
241	ADGRV1	1
242	CTTN	1
243	EPHB4	1
244	CHD4	1
245	MAP3K9	1
246	CDH9	1
247	AHNAK	1
248	NUMA1	1
249	MKI67	1
250	ABCC3	1
251	ABCC1	1
252	PRKDC	1
253	MUC16	1
254	NRG1	1
255	TIAM1	1
256	CEP290	1
257	KLRC2	1
258	MAP3K1	1
259	NBAS	1
260	PTPRB	1

261	TAF1	2
262	DDX3X	3
263	JAK1	2
264	LAMA2	2
265	ABCC2	1
266	CHD7	1
267	ERBB3	1
268	H3F3A	1
269	INPP4B	1
270	ROS1	1
271	SOS1	1
272	FLT3	1
273	KIT	1
274	RPS6KB1	1
275	PLXNA1	1
276	PLEC	1
277	VPS8	1
278	TACC2	1
279	RYR3	1
280	OTOG	1
281	KIAA1109	1
282	JRK	1
283	FBN3	1
284	DNAH14	1
285	CUX1	1
286	BIRC5	1
287	AK5	1
288	SMARCAL1	1
289	PIK3R1	1
290	IQCG	1
291	METTL24	1
292	IFI27	1
293	HLA-B	1
294	FGF2	1
295	FGF8	1
296	MED1	1
297	IRF6	1
298	EZH2	1
299	RIPK4	1
300	DICER1	1
301	BCL2L1	1
302	HPV 16	1
303	EVER 1	1
304	EVER 2	1
305	P53	4
306	XRCC1	1
307	XRCC3	1
308	XPC	1
309	XPD	1
310	IGF1	1
311	IGF2	1
312	HER2	1
313	HER3	1
314	PHF2	1
315	FANCC	1
316	CDKM2C	1
317	WNT10B	1
318	CEBPA	1
319	MAP3K8	1
320	FGR	1
321	GATA1	1
322	OL4	1
323	PDGFA	1
324	E2F2	1
325	LOH	1
326	PIK3R2	1
327	CHEK2	1
328	ARID1A	1
329	FANCM	1
330	NOTCH 4	1
